# Synthesis and Biological Evaluation of Indole-2-Carboxamides with Potent Apoptotic Antiproliferative Activity as EGFR/CDK2 Dual Inhibitors

**DOI:** 10.3390/ph15081006

**Published:** 2022-08-16

**Authors:** Lamya H. Al-Wahaibi, Yaser A. Mostafa, Mostafa H. Abdelrahman, Ali H. El-Bahrawy, Laurent Trembleau, Bahaa G. M. Youssif

**Affiliations:** 1 Department of Chemistry, College of Sciences, Princess Nourah bint Abdulrahman University, Riyadh 11671, Saudi Arabia; 2 Pharmaceutical Organic Chemistry Department, Faculty of Pharmacy, Assiut University, Assiut 71526, Egypt; 3 Department of Pharmaceutical Organic Chemistry, Faculty of Pharmacy, Al-Azhar University, Assiut 71524, Egypt; 4 Department of Pharmacology and Toxicology, Faculty of Pharmacy, Al-Azhar University, Assiut 71524, Egypt; 5 School of Natural and Computing Sciences, University of Aberdeen, Meston Building, Aberdeen AB24 3UE, UK

**Keywords:** indole, carboxamide, apoptosis, antiproliferative, multi-target

## Abstract

The apoptotic antiproliferative actions of our previously reported CB1 allosteric modulators 5-chlorobenzofuran-2-carboxamide derivatives **VIIa**–**j** prompted us to develop and synthesise a novel series of indole-2-carboxamide derivatives **5a**–**k**, **6a**–**c**, and **7**. Different spectroscopic methods of analysis were used to validate the novel compounds. Using the MTT assay method, the novel compounds were examined for antiproliferative activity against four distinct cancer cell lines. Compounds **5a**–**k**, **6a**–**c**, and **7** demonstrated greater antiproliferative activity against the breast cancer cell line (MCF-7) than other tested cancer cell lines, and **5a**–**k** (which contain the phenethyl moiety in their backbone structure) demonstrated greater potency than **6a**–**c** and **7**, indicating the importance of the phenethyl moiety for antiproliferative action. Compared to reference doxorubicin (GI_50_ = 1.10 µM), compounds **5d**, **5e**, **5h**, **5i**, **5j**, and **5k** were the most effective of the synthesised derivatives, with GI_50_ ranging from 0.95 µM to 1.50 µM. Compounds **5d**, **5e**, **5h**, **5i**, **5j**, and **5k** were tested for their inhibitory impact on EGFR and CDK2, and the results indicated that the compounds tested had strong antiproliferative activity and are effective at suppressing both CDK2 and EGFR. Moreover, the studied compounds induced apoptosis with high potency, as evidenced by their effects on apoptotic markers such as Caspases 3, 8, 9, Cytochrome C, Bax, Bcl2, and p53.

## 1. Introduction

In response to increased global morbidity and mortality rates from so-called incurable diseases, medication research and development has never been static but has grown increasingly dynamic. Traditionally, therapeutic drug discovery has relied on the design of highly selective chemical entities that target a single biological entity assumed to play a dominant role in a particular disease [1,2]. By means of this method, researchers hoped to eliminate any unwanted side effects and ensure that drug candidates had more drug-like properties. Highly selective or specific therapeutic medicines focused on single molecular targets, on the other hand, have shown to be ineffective, particularly in the treatment of complicated disorders. Drug resistance has been linked to the use of highly selective therapeutic agents.

However, due to the low efficacy of single target medications against multifactorial disorders whose aetiology is based on a collection of biochemical processes and many bioreceptors acting concurrently, drug design methodologies have to be reconsidered. Over the last few years, medicinal chemistry has been exploring new tools and alternatives to attain more agility, security, and efficiency in the synthesis and prospection of drug candidates.

Because of the ineffectiveness of certain single-drug therapies, the hunt for improved clinical outcomes has prompted the introduction of polypharmacology as a novel therapeutical technique. Polypharmacology is the development or application of pharmacological drugs that operate on several molecular targets or metabolic pathways. This multi-target strategy can take several forms, such as drug associations, drug combinations, or a single agent with numerous ligands, all of which are aimed at multiple targets. HIV/AIDS treatment, cancer treatment, TB treatment, and hypertension medication are all examples of combination therapy [3,4,5]. However, the inefficiency of combination therapy, as well as the negative consequences of drug–drug interactions, different pharmacokinetics, toxicity, and costs, has fuelled the development of new drug discovery methodologies. This current technique promotes combining diverse structural components in a single scaffold to allow molecular recognition by more than one bioreceptor, operating in many targets associated with biochemical networks responsible for multifactorial disease pathophysiology [6,7,8].

A breakdown of balance between cell proliferation and apoptosis is a symptom that enhances the inability of damaged cells to be removed by apoptosis. Activating apoptotic pathways in tumour cells is a critical practise for cancer treatment [9]. Apoptosis is triggered by extracellular or intracellular cues, which initiate a signalling cascade with characteristics such as nuclear condensation and DNA fragmentation [10]. Furthermore, the deregulations responsible for cancer genesis and progression involve hundreds of genes or signalling cascades [11]

Caspase, a highly specialized family of cysteine proteases, is known to mediate an important stage of the apoptotic process [12]. Numerous in vitro and in vivo research demonstrated that aberrant caspase activation control is critical to avoiding cancer cell death [13]. Furthermore, other genes, including Bcl-2 and p53, are known to be involved in apoptotic pathways. Overexpression of anti-apoptotic Bcl-2 has been linked to a variety of cancers [14]. The suppression of caspase proteins is thought to be the mechanism by which Bcl-2 prevents apoptosis [15]. p53 has been discovered to be required for cellular senescence caused by mutations in genes involved in mitosis and chromosomal segregation [16]. To maintain genomic integrity, the p53 gene can activate cell cycle checkpoints, DNA repair, and apoptosis (Ahmad et al., 2012). Most malignancies are caused by p53 mutations or deletions [17].

**Figure 1:**
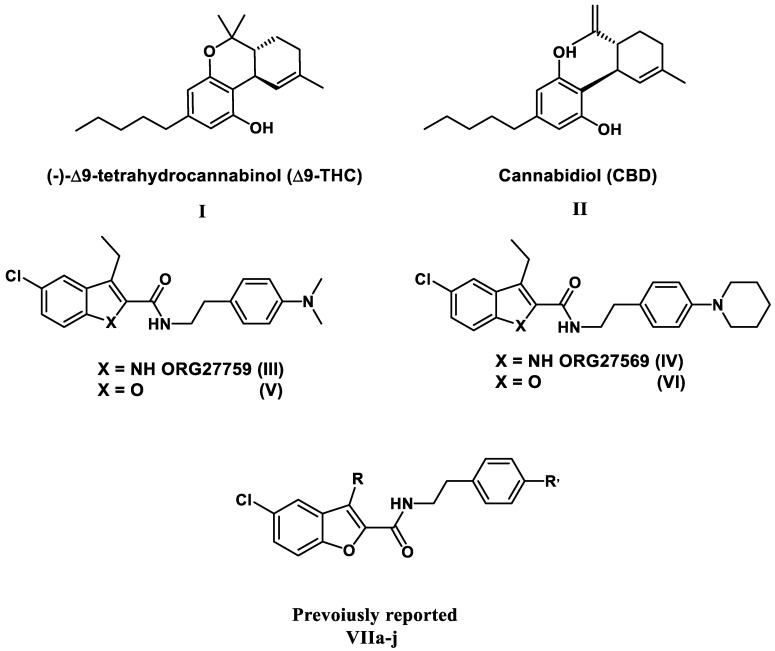
Structures of compounds **I**–**VI** and **VIIa**–**j**.

Antiproliferative actions have been documented for cannabinoids such as THC (**I**) and the CB1 allosteric modulator CBD (**II**) [18,19,20,21,22], but no data for other CB1 allosteric modulators such as the 5-chloroindole-2-carboxamide derivatives **III** and **IV**, and their furan congeners **V** and **VI** have been reported (Figure 1). Recently, we reported on the antiproliferative action of 5-chlorobenzofuran-2-carboxamide CB1 allosteric modulators **V** and **VI** for the first time [23]. Based on this, we designed and synthesised a novel series of 5-chlorobenzofuran-2-carboxamide derivatives **VIIa**–**j** (Figure 1). The newly synthesized compounds were tested for their antiproliferative effects in A549 lung, MCF-7 breast, Panc-1 Pancreatic, and HT-29 colon cancer cell lines. **VIIa**–**j** compounds showed significant antiproliferative action, the most potent derivative of **VIIa**–**j** had a GI_50_ value of 1.35 µM against the four examined cell lines, being equipotent to the reference doxorubicin (mean GI_50_ = 1.13 µM) and even more potent than doxorubicin in MCF-7. The compounds examined had a strong apoptotic effect, with significant increases in caspase 3, 8, and 9, as well as Cytochrome C levels. Furthermore, compared to doxorubicin, the investigated compounds triggered a significant rise in Bax levels and a decrease in anti-apoptotic Bcl-2 protein levels in MCF-7 cells [23].

**Figure 2:**
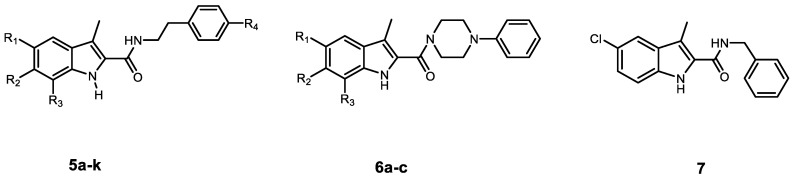
Structures of new compounds **5a**–**k**, **6a**–**c**, and **7**.

In the present work, the apoptotic antiproliferative actions of our previously reported 5-chlorobenzofuran-2-carboxamide derivatives **VIIa**–**j** (Figure 1) prompted us to develop and synthesise a novel series of indole-2-carboxamide derivatives **5a**–**k**, **6a**–**c**, and **7** (Figure 2). A small library of fifteen new compounds in which the methyl group was kept as a substituent at C3. To investigate the effect of substituent modification, the *para* positions of the phenethyl tails in the newly synthesised compounds were left unsubstituted or substituted with 4-dimethylamino, morpholin-4-yl, piperidin-1-yl, or 2-methylpyrrolidine-1-yl. To investigate the impact of the linker nature on anticancer activity, the phenethyl amino carbonyl moieties were modified to 4 phenylpiperazin-1-yl carbonyl as in compounds **6a**–**c** or benzyl carbonyl as in compound **7**. The position and number of halogen atoms on the indole moiety’s phenyl ring were also investigated. The antiproliferative activity of compounds **5a**–**k**, **6a**–**c**, and **7** against a panel of cancer cell lines were investigated. The most active compounds were evaluated for mechanistic activity as multi-targeted kinase inhibitors such as EGFR and CDK2. Furthermore, the compounds were evaluated for apoptotic activity against caspases 3, 8, and 9, as well as Cytochrome C, Bax, Bcl2, and p53.

## 2. Results and Discussion

### 2.1. Chemistry

**Scheme 1:**
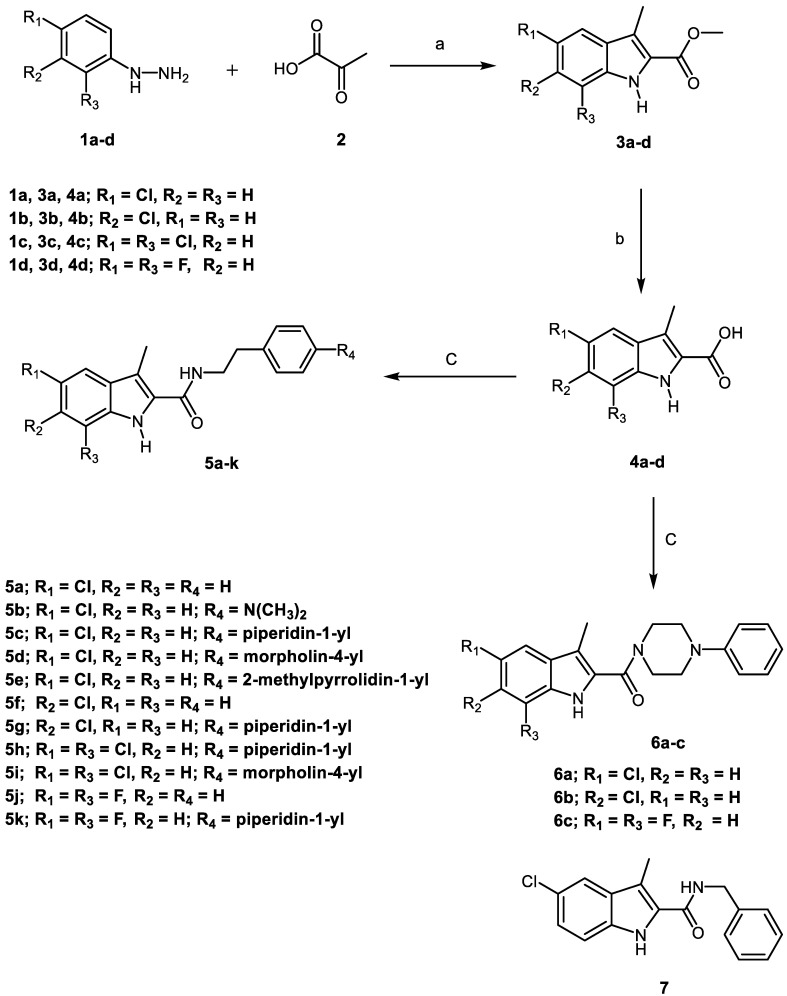
Synthesis of the target compounds **5a**–**k**, **6a**–**c**, and **7.** Reagents and conditions: (a) PTSA, EtOH, reflux, 20 h, 82%; (b) 5% NaOH, EtOH, 40 °C, overnight, 95%; (c) BOP, DIPEA, DCM, rt, overnight, 75–94%.

Scheme 1 depicts the synthesis of target compounds **5a**–**k**, **6a**–**c**, and **7**. Derivatives of phenyl hydrazine Hydrochloride **1a**–**d** under Fisher Indole cyclization were reacted with 2-oxopropanoic acid **2** in the presence of PTSA (*p*-toluenesulfonic acid) to provide 3-methylindole-2-carboxylates **3a**–**d** [24]. The carboxylic acids **4a**–**d** was obtained by alkaline hydrolysis of the esters **3a**–**d** [25]. The appropriate amines were coupled with carboxylic acids **4a**–**d** in the presence of DIPEA in DCM using BOP as a coupling reagent [23], yielding target carboxamides **5a**–**k**, **6a**–**c**, and **7**. ^1^H NMR, ^13^C NMR, and HRESI-MS were used to identify the newly synthesised derivatives. The ^1^H NMR spectrum of **5d** revealed the appearance of three singlet signals: one at δ 11.34 ppm (1H) consistent with indole NH, one at δ 7.89 ppm (1H) relating to amidic NH, and one at δ 2.41 ppm (3H) corresponding to the methyl group. The spectrum also indicated the existence of signals corresponding to ethylene protons at δ 3.48 (q, *J* = 7.1 Hz, 2H, NHCH_2_) and δ 2.77 (t, *J* = 7.4 Hz, 2H, NHCH_2_), in addition to the morpholine group’s distinctive signals at δ 3.70 (t, *J* = 4.8 Hz, 4H, morph-H) and δ 3.02 (t, *J* = 4.8 Hz, 4H, morph-H). HRESI-MS revealed a peak for [M + H]^+^ at *m*/*z* 398.1629, which corresponds to the molecular formula C_22_H_25_ClN_3_O_2_.

### 2.2. Evaluation of Biological Activities

#### 2.2.1. In Vitro Anticancer Activity

##### Cell Viability Assay

The MCF-10A (human mammary gland epithelial) cell line was used in the cell viability experiment. Compounds **5a**–**k**, **6a**–**c**, and **7** were incubated with MCF-10A cells for 4 days at 50 µM concentration, and the viability of cells was determined using the 3-(4,5-dimethylthiazol-2-yl)-2,5-diphenyltetrazolium bromide (MTT) test. [26]. All compounds had no cytotoxic effects, and the vitality of the cells was more than 83% for most of the compounds examined.

##### Antiproliferative Activity

Using the MTT assay with doxorubicin as the reference drug, the antiproliferative activities of **5a**–**k**, **6a**–**c**, and **7** against four human cancer cell lines, including pancreas cancer cell line (Panc-1), breast cancer cell line (MCF-7), colon cancer cell line (HT-29), and epithelial cancer cell line (A-549) were investigated [27]. Table 1 shows the results of calculating the median inhibitory concentration (IC_50_) for all derivatives. Generally, compounds **5a**–**k**, **6a**–**c**, and **7** demonstrated greater antiproliferative activity against the breast cancer cell line (MCF-7) than other tested cancer cell lines, and **5a**–**k** (which contain the phenethyl moiety in their backbone structure) demonstrated greater potency than **6a**–**c** and **7**, indicating the importance of the phenethyl moiety for antiproliferative action.

The 2-methylpyrrolidin-4-yl phenethyl derivative **5e** (R_1_ = Cl, R_2_ = R_3_ = H, R_4_ = 2-methylpyrrolidin-1-yl) was the most potent derivative, with a GI_50_ value of 0.95 µM against the four cell lines, being more potent than the reference doxorubicin (GI_50_ = 1.10 µM) and also it was more potent than doxorubicin in A-549, MCF-7, and Panc-1 cell lines (IC_50_ = 0.95, 0.80, and 1.00 µM, respectively, while for doxorubicin IC_50_ = 1.20, 0.90, and 1.40 µM, respectively). The unsubstituted derivative **5a** (R_1_ = Cl, R_2_ = R_3_ = R_4_ = H) was roughly four-fold less effective than **5e**, with a GI_50_ = 3.70 µM, whereas the 4-dimethylamino derivative **5b** (R_1_ = Cl, R_2_ = R_3_ = H, R_4_ = dimethylamino) had a GI_50_ = 3.30 µM.

Compound **5d** (R_1_ = Cl, R_2_ = R_3_ = H, R_4_ = morpholin-4-yl) rated second in activity with a GI_50_ of 1.05 µM against the four cancer cell lines, being somewhat less potent (1.1-fold) than **5e** but equipotent to doxorubicin and even more potent than doxorubicin against A-549 and Panc-1 cell lines.

Replacement of the 2-methylpyrrolidin-4-yl moiety in compound **5e** or the morpholin-4-yl in **5d** by 4-piperidin-1-yl in compound **5c** resulted in at least 1.8- and 1.6-fold reduction of the mean GI_50_ values, respectively, signifying the importance of the 2-methylpyrrolidin-4-yl and 4-morpholinophenethyl moieties for the antiproliferative activity.

The 5-substitution impact was also investigated. As indicated in Table 1, compound **5g** (R_2_ = Cl, R_1_ = R_3_ = H, R_4_ = 4-piperidin-1-yl) had much lower antiproliferative efficacy (3 times) than compound **5c** (R_1_ = Cl, R_2_ = R_3_ = H, R_4_ = 4-piperidin-1-yl). Compound **5f** (R_2_ = Cl, R_1_ = R_3_ = H = R_4_ = H) was, on the other hand, more potent (1.9 times) than compound **5a** (R_1_ = Cl, R_2_ = R_3_ = R_4_ = H).

Furthermore, we attempt to explore the effect of increasing the number of halogen atoms on antiproliferative activity. For instance, the dihalo derivatives **5h** (R_1_ = R_3_ = Cl, R_2_ = H, R_4_ = 4-piperidin-1-yl) and **5k** (R_1_ = R_3_ = F, R_2_ = H, R_4_ = 4-piperidin-1-yl) had higher antiproliferative activity than the monohalo derivative **5c** (R_1_ = Cl, R_2_ = R_3_ = H, R_4_ = 4-piperidin-1-yl) with GI_50_ values of 1.10 µM and 1.40 µM, respectively, compared to **5c** (GI_50_ = 1.70 µM), indicating the relevance of dihalo atoms for antiproliferative activity and that the chlorine atom is better tolerated than the fluorine one. The same is true for **5j** (R_1_ = R_3_ = F, R_2_ = R_4_ = H), which has higher potency (GI_50_ = 1.20 µM) than **5a** (R_1_ = Cl, R_2_ = R_3_ = R_4_ = H, GI_50_ = 3.7 µM) and **5f** (R_2_ = Cl, R_1_ = R_3_ = H = R_4_ = H, GI_50_ = 1.95 µM). The situation is somewhat different in the case of 5,7-dichloro derivative **5i** (R_1_ = R_3_ = Cl, R_2_ = H, R_4_ = morpholin-4-yl), which demonstrated lower potency (GI_50_ = 1.50 µM) than the 5-chloro derivative **5d** (R_1_ = Cl, R_2_ = R_3_ = R_4_ = morpholin-4-yl) with a GI_50_ value of 1.05 µM.

Furthermore, among the tested compounds, the 4-benzyl carbonyl derivative **7** and the 4-phenylpiperazin-1-yl carbonyl derivatives **6a**–**c** had the lowest mean GI_50_ values, implying that the *N*-phenethyl carboxamide architecture is important for antiproliferative action and correlating with previous SAR studies [23].

#### 2.2.2. EGFR Inhibitory Activity

The inhibitory efficacy of **5d**, **5e**, and **5h**–**k** against EGFR was evaluated using the EGFR-TK assay [28], and the findings are presented in Table 2. The results of this test supplement the findings of the cancer-cell-based investigation. All the tested derivatives (**5d**, **5e**, and **5h**–**k**) inhibited EGFR significantly, with IC_50_ values ranging from 89 to 137 nM. Based on the findings given, three derivatives **5d**, **5e**, and **5j** were found to be the most potent, with EGFR inhibitory effects (IC_50_ = 89 ± 6 nM and 93 ± 8 nM, and 98 ± 8 nM, respectively) comparable to the positive erlotinib (IC_50_ = 80 ± 5 nM). Again, the 2-methylpyrrolidin-1-yl phenethyl derivative **5e** (R_1_ = Cl, R_2_ = R_3_ = H, R_4_ = 2-methylpyrrolidin-1-yl) and the 4-morpholin-4-yl phenethyl 5d (R_1_ = Cl, R_2_ = R_3_ = H, R_4_ = morpholin-4-yl) were the most potent of all synthesized derivatives, with IC_50_ value of 93 nM and 89 nM being equipotent to the reference erlotinib.

#### 2.2.3. CDK2 Inhibitory Assay

Compounds **5d**, **5e**, and **5h**–**k** were further examines for their ability to inhibit the CDK2 enzyme [29]. The IC_50_ values are shown in Table 2. In comparison to the reference dinaciclib (IC_50_ = 20 nM), all investigated derivatives inhibited CDK2 effectively, with IC_50_ values ranging from 11 nM to 34 nM. Three derivatives, **5e**, **5h**, and **5k**, were shown to be superior to the standard dinaciclib as CDK2 inhibitors, with IC_50_ values of 13, 11, and 19 nM, respectively. Compound **5e**, the most potent antiproliferative derivative, displayed significant anti-CDK2 activity with an IC_50_ value of 13 nM, which is 1.5-fold more active than the reference dinaciclib. On the other hand, compounds **5d**, **5i**, **5j**, and **5k** exhibited significant activity against CDK2 (IC_50_ = 23, 27, 34, and 19 µM) comparable to dinaciclib. The findings of the EGFR and CDK2 tests revealed that the examined compounds exhibit significant antiproliferative activity and are efficient at suppressing both CDK2 and EGFR.

#### 2.2.4. Apoptosis Assay

A previous report has shown that **CBD** (**2**), a CB1 allosteric modulator, can trigger apoptosis [30]. Therefore, to assess the proapoptotic potential of our target compounds, we evaluated the most active compounds **5d**, **5e**, and **5h** for their capacity to initiate the apoptosis cascade in the breast cancer (MCF-7) cell line.

##### Activation of Proteolytic Caspases Cascade

Caspases play a crucial role in the initiation and completion of the apoptotic process [31]. Caspase-3 is a crucial caspase that cleaves a variety of proteins in cells, causing apoptosis [32]. The effects of compounds **5d**, **5e**, and **5h** on caspase 3 were assessed and compared to doxorubicin, which was used as a control [33]. The results showed that when compared to control cells, the tested compounds increased the level of active caspase 3 by 8–10 folds and that **5d**, **5e**, and **5h** induce outstanding overexpression of caspase-3 protein level (570.00 ± 5.00, 635.50 ± 5.50 and 537.50 ± 5.00 pg/mL, respectively) compared to doxorubicin (503.50 ± 4.50 pg/mL). In comparison to the control untreated cells, the most active antiproliferative derivative **5e** increases caspase 3 levels by 9.70 times.

The impact of compounds **5d**, **5e**, and **5h** on caspases 8 and 9 was also investigated to highlight the involvement of the intrinsic and extrinsic apoptotic pathways in the antiproliferative actions of these compounds, Table 3. When compared to control cells, compound **5e** increased caspase 8 and 9 levels by 10.90 and 18.15 folds, respectively, while compound **5d** increased caspase 8 and 9 levels by 9.70 and 17.80 folds, respectively, indicating activation of both intrinsic and extrinsic pathways with a stronger effect on the intrinsic pathway because caspase 9 levels were higher [34].

##### Cytochrome C Assay

The quantity of cytochrome C within the cell is important for activating caspases and commencing the intrinsic apoptosis process [35]. Table 3 shows the findings of testing indole-2-carboxamide derivatives **5d**, **5e**, and **5h** as Cytochrome C activators in the MCF-7 human breast cancer cell line. Compounds **5d**, **5e**, and **5h** increased Cytochrome C levels in the MCF-7 human breast cancer cell line by 14, 16, and 13 times, respectively, compared to untreated control cells. The findings add to the evidence that apoptosis can be attributed to Cytochrome C overexpression and activation of the intrinsic apoptotic pathway triggered by the investigated compounds.

##### Bax and Bcl-2 Levels Assay

The most potent caspase activators, **5d** and **5e**, were investigated further for their influence on Bax and Bacl-2 levels in a breast cancer cell line (MCF-7) using doxorubicin as a control [36]. Table 4 shows that **5d** and **5e** caused a significant increase in Bax levels when compared to doxorubicin. Compound **5e** demonstrated a comparable induction of Bax (296.50 pg/mL) compared to doxorubicin (276 pg/mL) with a 36-fold increase over control untreated breast cancer cells, followed by compound 5d (290 pg/mL and 35-fold rise). Finally, compound **5e** reduced the anti-apoptotic Bcl-2 protein levels to 0.87 ng/mL in MCF-7 cells, followed by compound **5d** (0.89 ng/mL) in comparison to doxorubicin (0.98 ng/mL).

##### Effect of Compounds **5d** and **5e** on p53 Transcription in MCF-7

p53 is a unique protein that participates in several physiological processes such as cell metabolism [37], stem cell maintenance [38], and cell adhesion [39]. Because p53 is frequently inactivated in cancer cells, the cells are unable to undergo apoptosis [40,41]. Similarly, activating, or stabilizing p53 aids cancer cells in normalizing p53-controlled physiological processes and increasing apoptotic activity [42]. The effects of **5d** and **5e** on p53 transcription were evaluated and compared to doxorubicin as a control [43], Table 5. The results revealed an increase of at least 27-folds in p53 level compared to the test cells and that the p53 protein level of **5d** and **5e** was significantly inductive (1375 and 1435 pg/mL, respectively) in relation to doxorubicin (1265 pg/mL).

### 2.3. Docking Study

**Figure 3:**
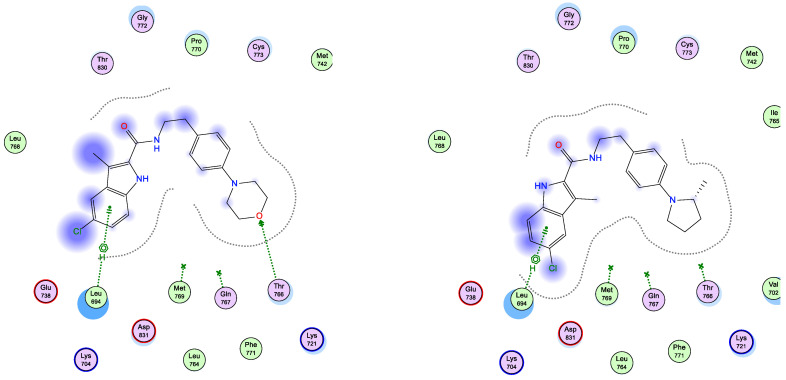
Schematic 2D representation of best docking poses of **5d** (**left**) and **5e** (**right**) within EGFR (PDB ID: 1M17) active site showing pi-H (green-dotted line) and H-acceptor interactions (green arrow).

Interestingly, running docking simulations of compounds **5d** and **5e** within EGFR active site revealed docking scores (S; −6.90 and −6.79 kcal/mol; respectively), so much close to that of co-crystallized ligand, erlotinib (−7.30 kcal/mol), which co-insides with what obtained in-vitro against EGFR enzyme (as shown in Table 2). Moreover, visual inspection of the best docking poses of compounds **5d** and **5e** showed their close distance to key amino acids lining EGFR active site. Additionally, compounds **5d** and **5e** showed a number of H-bonding and pi–H interactions with LEU 694 and THR 766 amino acid residues (as listed in Table 6 and shown in Figure 3).

Both compounds **5d** and **5e** showed a common settling profile within EGFR active represented by U-shaped bending of the whole molecule, so its indole ring interacts with LEU 694 (as shown in Figure 3). On the other hand, compound **5d** showed additional H-acceptor bonding with THR 766 that resulted in its better docking score over its congener, compound **5e**.

**Figure 4:**
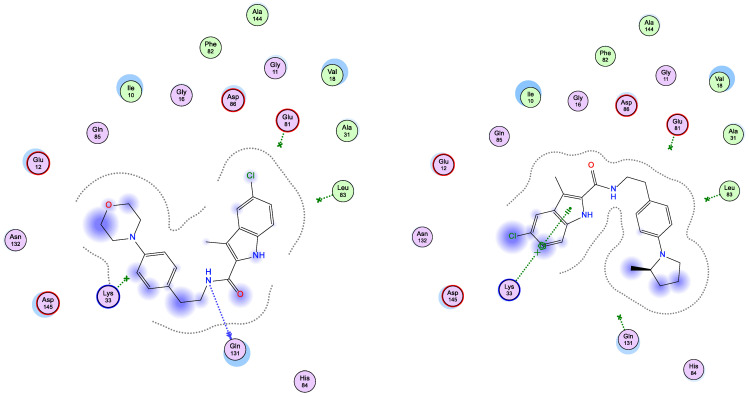
Schematic 2D representation of best docking poses of **5d** (**left**) and **5e** (**right**) within CDK2 (PDB ID: 1PYE) active site showing pi-cation (green-dotted line) and H-donor interactions (blue arrow).

Additionally, and as shown in Table 6, MDs of compound **5e** within the CDK2 active site revealed its better docking score (S = −6.99 kcal/mol) over its congener **5d** (S = −6.03 kcal/mol), although its inability to have strong H-bonding with amino acid residues lining active site, its close proximity to key amino acid residues (revealed by its proximity contour as shown in Figure 4) could explain its better scoring over **5d**.

## 3. Materials and Methods

### 3.1. Chemistry

3-Methylindole-2-carboxylates **3a**–**d** [24], carboxylic acids **4a**–**d** [25], and carboxamides **5a**–**k**, **6a**–**c**, and **7** [23] were synthesized according to previously reported procedures.

#### 3.1.1. 5-Chloro-3-methyl-N-phenethyl-1H-indole-2-carboxamide (**5a**)

Yield % 91, m.p 182–184 °C, ^1^H NMR (400 MHz, CDCl_3_) δ 9.21 (s, 1H, indole NH), 7.53 (d, *J* = 2.2 Hz, 1H, Ar-H), 7.39–7.17 (m, 7H, Ar-H), 5.97 (s, 1H, amide NH), 3.81 (q, *J* = 6.4 Hz, 2H, NHCH_2_), 2.97 (t, *J* = 6.8 Hz, 2H, NHCH_2_CH_2_), 2.26 (s, 3H, CH_3_). ^13^C NMR (101 MHz, CDCl_3_) δ 162.10 (C=O), 138.50, 133.32, 128.85, 128.81, 128.53, 126.84, 125.53, 125.00, 119.38, 112.79, 110.80, 40.83, 35.51, 9.88. HRESI-MS *m*/*z* calcd for [M + H]^+^ C_18_H_18_ClN_2_O: 313.1102, found: 313.1104.

#### 3.1.2. 5-Chloro-N-(4-(dimethylamino)phenethyl)-3-methyl-1H-indole-2-carboxamide (**5b**)

Yield % 89, m.p 202–204 °C. ^1^H NMR (400 MHz, DMSO-*d*_6_) δ 11.34 (s, 1H, indole NH), 7.87 (t, *J* = 5.6 Hz, 1H, amide NH), 7.62 (d, *J* = 2.0 Hz, 1H, Ar-H), 7.37 (d, *J* = 8.6 Hz, 1H, Ar-H), 7.16 (dd, *J* = 8.7, 2.1 Hz, 1H, Ar-H), 7.06 (d, *J* = 8.6 Hz, 2H, Ar-H), 6.66 (d, *J* = 8.7 Hz, 2H, Ar-H), 3.46 (q, *J* = 6.7 Hz, 2H, NHCH_2_), 2.82 (s, 6H, N(CH_3_)_2_), 2.74 (t, *J* = 6.6 Hz, 2H, NHCH_2_CH_2_), 2.41 (s, 3H, CH_3_). ^13^C NMR (101 MHz, DMSO-*d*_6_) δ 162.01 (C=O), 149.54, 134.07, 129.94, 129.59, 129.55, 127.31, 124.06, 123.97, 119.35, 113.92, 113.13, 112.93, 41.47, 40.79, 34.69, 9.98. HRESI-MS *m*/*z* calcd for [M + H]^+^ C_20_H_23_ClN_3_O: 356.1524, found: 356.1522.

#### 3.1.3. 5-Chloro-3-methyl-N-(4-(piperidin-1-yl)phenethyl)-1H-indole-2-carboxamide (**5c**)

Yield % 92, m.p 216–218 °C, ^1^H NMR (400 MHz, CDCl_3_) δ 9.17 (s, 1H, indole NH), 7.53 (s, 1H, Ar-H), 7.29 (d, *J* = 8.6 Hz, 1H, Ar-H), 7.20 (dd, *J* = 8.6, 0.7 Hz, 1H, Ar-H), 7.13 (d, *J* = 8.6 Hz, 2H, Ar-H), 6.92 (d, *J* = 8.6 Hz, 2H, Ar-H), 5.97 (s, 1H, amide NH), 3.76 (q, *J* = 6.6 Hz, 2H, NHCH_2_), 3.17–3.09 (m, 4H, piperidin-H), 2.87 (t, *J* = 6.7 Hz, 2H, NHCH_2_CH_2_), 2.27 (s, 3H, CH_3_), 1.74–168 (m, 4H, piperidin-H), 1.63–1.53 (m, 2H, piperidin-H). ^13^C NMR (101 MHz, CDCl_3_) δ 162.03 (C=O), 151.21, 133.26, 129.74, 129.37, 128.77, 128.68, 125.48, 124.90, 119.37, 116.95, 112.75, 110.71, 50.82, 40.92, 34.48, 25.79, 24.25, 9.94. HRESI-MS *m*/*z* calcd for [M + H]^+^ C_23_H_27_ClN_3_O: 396.1837, found: 396.1837.

#### 3.1.4. 5-Chloro-3-methyl-N-(4-morpholinophenethyl)-1H-indole-2-carboxamide (**5d**)

Yield % 91, m.p 210–212 °C. ^1^H NMR (400 MHz, DMSO-*d*_6_) δ 11.34 (s, 1H, indole NH), 7.89 (t, *J* = 5.6 Hz, 1H, amide NH), 7.62 (s, 1H, Ar-H), 7.38 (d, *J* = 8.6 Hz, 1H, Ar-H), 7.21–7.08 (m, 3H, Ar-H), 6.85 (d, *J* = 8.3 Hz, 2H, Ar-H), 3.70 (t, *J* = 4.8 Hz, 4H, morph-H), 3.48 (q, *J* = 7.1 Hz, 2H, NHCH_2_), 3.02 (t, *J* = 4.8 Hz, 4H, morph-H), 2.77 (t, *J* = 7.4 Hz, 2H, NHCH_2_), 2.41 (s, 3H, CH_3_). ^13^C NMR (101 MHz, DMSO-*d*_6_) δ 162.05 (C=O), 149.99, 134.08, 130.44, 129.91, 129.62, 129.59, 124.08, 124.00, 119.36, 115.72, 113.94, 112.98, 66.57, 49.17, 41.29, 34.71, 9.98. HRESI-MS *m*/*z* calcd for [M + H]^+^ C_22_H_25_ClN_3_O_2_: 398.1630, found: 398.1629.

#### 3.1.5. 5-Chloro-3-methyl-N-(4-(2-methylpyrrolidin-1-yl)phenethyl)-1H-indole-2-carboxamide (**5e**)

Yield % 85, m.p 186–188 °C. ^1^H NMR (400 MHz, CDCl_3_) δ 9.67 (s, 1H, indole NH), 7.53 (d, *J* = 2.0 Hz, 1H, Ar-H), 7.32 (d, *J* = 8.6 Hz, 1H, Ar-H), 7.20 (dd, *J* = 8.7, 2.0 Hz, 1H, Ar-H), 7.10 (d, *J* = 8.5 Hz, 2H, Ar-H), 6.57 (d, *J* = 8.5 Hz, 2H, Ar-H), 6.07 (t, *J* = 5.5 Hz, 1H, amide NH), 3.91–3.71 (m, 3H, pyrrolidin-H, NHCH_2_), 3.47–3.37 (m, 1H, pyrrolidin-H), 3.18–3.12 (m, 1H, pyrrolidin-H), 2.87 (t, *J* = 6.7 Hz, 2H, NHCH_2_CH_2_), 2.31 (s, 3H, CH_3_), 2.15–1.94 (m, 3H, pyrrolidin-H), 1.73–1.70 (m, 1H, pyrrolidin-H), 1.17 (d, *J* = 6.2 Hz, 3H, CHCH_3_). ^13^C NMR (101 MHz, CDCl_3_) δ 162.23 (C=O), 146.18, 133.48, 129.68, 129.54, 128.78, 125.34, 124.78, 124.45, 119.29, 112.92, 112.16, 110.77, 53.67, 48.26, 41.25, 34.42, 33.09, 23.28, 19.29, 10.01. HRESI-MS *m*/*z* calcd for [M + H]^+^ C_23_H_27_ClN_3_O: 396.1837, found: 396.1832.

#### 3.1.6. 6-Chloro-3-methyl-N-phenethyl-1H-indole-2-carboxamide (**5f**)

Yield % 80, m.p 170–172 °C, ^1^H NMR (400 MHz, CDCl_3_) δ 9.67 (s, 1H, indole NH), 7.46 (d, *J* = 8.6 Hz, 1H, Ar-H), 7.42–7.22 (m, 6H, Ar-H), 7.07 (dd, *J* = 8.6, 1.9 Hz, 1H, Ar-H), 6.00 (s, 1H, amide NH), 3.82 (q, *J* = 6.4 Hz, 2H, NHCH_2_), 2.98 (t, *J* = 6.7 Hz, 2H, NHCH_2_CH_2_), 2.28 (s, 3H, CH_3_). ^13^C NMR (101 MHz, CDCl_3_) δ 162.39 (C=O), 138.53, 135.49, 130.42, 128.84, 128.83, 127.88, 127.20, 126.81, 120.92, 120.67, 111.61, 40.89, 35.53, 9.94. HRESI-MS *m*/*z* calcd for [M + H]^+^ C_18_H_18_ClN_2_O: 313.1102, found: 313.1101.

#### 3.1.7. 6-Chloro-3-methyl-N-(4-(piperidin-1-yl)phenethyl)-1H-indole-2-carboxamide (**5g**)

Yield % 75, m.p 218–220 °C, ^1^H NMR (400 MHz, CDCl_3_) δ 9.19 (s, 1H, indole NH), 7.47 (d, *J* = 8.6 Hz, 1H, Ar-H), 7.37 (d, *J* = 1.8 Hz, 1H, Ar-H), 7.13 (d, *J* = 8.6 Hz, 2H, Ar-H), 7.08 (dd, *J* = 8.6, 1.8 Hz, 1H, Ar-H), 6.92 (d, *J* = 8.6 Hz, 2H, Ar-H), 5.97 (s, 1H, amide NH), 3.76 (q, *J* = 6.6 Hz, 2H, NHCH_2_), 3.17–3.09 (m, 4H, piperidin-H), 2.88 (t, *J* = 6.6 Hz, 2H, NHCH_2_CH_2_), 2.29 (s, 3H, CH_3_), 1.76–1.66 (m, 4H, piperidin-H), 1.64–1.54 (m, 2H, piperidin-H). ^13^C NMR (101 MHz, CDCl_3_) δ 162.12 (C=O), 151.17, 135.22, 130.41, 129.39, 128.87, 128.04, 127.32, 120.95, 120.70, 116.97, 111.44, 111.39, 50.86, 40.93, 34.51, 25.79, 24.24, 9.97. HRESI-MS *m*/*z* calcd for [M + H]^+^ C_23_H_27_ClN_3_O: 396.1837, found: 396.1837.

#### 3.1.8. 5,7-Dichloro-3-methyl-N-(4-(piperidin-1-yl)phenethyl)-1H-indole-2-carboxamide (**5h**)

Yield % 85, m.p 178–180 °C, ^1^H NMR (400 MHz, DMSO-*d*_6_) δ 11.43 (s, 1H, indole NH), 8.36 (t, *J* = 5.6 Hz, 1H, amide NH), 7.66 (d, *J* = 1.8 Hz, 1H, Ar-H), 7.36 (d, *J* = 1.9 Hz, 1H, Ar-H), 7.07 (d, *J* = 8.0 Hz, 2H, Ar-H), 6.83 (d, *J* = 8.3 Hz, 2H, Ar-H), 3.45 (q, *J* = 7.3 Hz, 2H, NHCH_2_), 3.03 (t, *J* = 5.4 Hz, 4H, piperidin-H), 2.75 (t, *J* = 7.5 Hz, 2H, NHCH_2_CH_2_), 2.46 (s, 3H, CH_3_), 1.63–1.43 (m, 6H, piperidin-H). ^13^C NMR (101 MHz, DMSO-*d*_6_) δ 161.24 (C=O), 150.70, 131.41, 130.65, 130.24, 129.68, 129.53, 124.16, 123.24, 118.73, 117.38, 116.60, 116.52, 50.33, 41.27, 34.69, 25.76, 24.34, 10.19. HRESI-MS *m*/*z* calcd for [M + H]^+^ C_23_H_26_Cl_2_N_3_O: 430.1447, found: 430.1448.

#### 3.1.9. 5,7-Dichloro-3-methyl-N-(4-morpholinophenethyl)-1H-indole-2-carboxamide (**5i**)

Yield % 84, m. p 185–187 °C. ^1^H NMR (400 MHz, CDCl_3_) δ 9.25 (s, 1H, indole NH), 7.43 (d, *J* = 1.7 Hz, 1H, Ar-H), 7.25 (d, *J* = 1.8 Hz, 1H, Ar-H), 7.15 (d, *J* = 8.5 Hz, 2H, Ar-H), 6.88 (d, *J* = 8.7 Hz, 2H, Ar-H), 6.02 (s, 1H, amide NH), 3.90–3.83 (m, 4H, morph-H), 3.77 (q, *J* = 6.6 Hz, 2H, NHCH_2_), 3.17–3.09 (m, 4H, morph-H), 2.89 (t, *J* = 6.7 Hz, 2H, NHCH_2_CH_2_), 2.28 (s, 3H, CH_3_). ^13^C NMR (101 MHz, CDCl_3_) δ 161.59 (C=O), 150.23, 130.98, 130.33, 129.69, 129.53, 129.33, 125.41, 123.94, 118.19, 117.65, 116.11, 111.86, 66.86, 49.47, 41.01, 34.48, 10.12. HRESI-MS *m*/*z* calcd for [M + H]^+^ C_22_H_24_Cl_2_N_3_O_2_: 432.1240, found: 432.1240.

#### 3.1.10. 5,7-Difluoro-3-methyl-N-phenethyl-1H-indole-2-carboxamide (**5j**)

Yield % 82, m.p 198–200 °C, ^1^H NMR (400 MHz, DMSO-*d*_6_) δ 11.95 (s, 1H, indole NH), 8.45 (t, *J* = 5.6 Hz, 1H, amide NH), 7.69–7.50 (m, 6H, Ar-H), 7.42 (t, *J* = 11.6 Hz, 1H, Ar-H), 3.86 (q, *J* = 7.8 Hz, 2H, NHCH_2_), 3.20 (t, *J* = 7.4 Hz, 2H, NHCH_2_CH_2_), 2.75 (s, 3H, CH_3_). ^13^C NMR (101 MHz, DMSO-*d*_6_) δ 161.91 (C=O), 140.15, 130.98, 129.46, 129.17, 126.95, 120.88, 116.06, 101.43, 101.15, 99.81, 99.61, 99.50, 41.29, 35.83, 10.49. HRESI-MS *m*/*z* calcd for [M + H]^+^ C_18_H_17_F_2_N_2_O: 315.1303, found: 315.1305.

#### 3.1.11. 5,7-Difluoro-3-methyl-N-(4-(piperidin-1-yl)phenethyl)-1H-indole-2-carboxamide (**5k**)

Yield % 88, m.p 192–194 °C, ^1^H NMR (400 MHz, DMSO-*d*_6_) δ 11.61 (s, 1H, indole NH), 8.07 (s, 1H, amide NH), 7.25 (dd, *J* = 9.3, 2.2 Hz, 1H, Ar-H), 7.13–7.04 (m, 3H, Ar-H), 6.84 (d, *J* = 8.6 Hz, 2H, Ar-H), 3.46 (q, *J* = 7.2 Hz, 2H, NHCH_2_), 3.05 (t, *J* = 5.4 Hz, 4H, piperidin-H), 2.74 (t, *J* = 7.4 Hz, 2H, NHCH_2_CH_2_), 2.42 (s, 3H, CH_3_), 1.64–1.55 (m, 4H, piperidin-H), 1.51–1.48 (m, 2H, piperidin-H). ^13^C NMR (101 MHz, DMSO-*d*_6_) δ 161.50 (C=O), 157.34, 155.00, 150.69, 147.63, 131.03, 129.65, 120.65, 116.52, 115.77, 101.08, 99.44, 99.24, 50.33, 41.24, 34.64, 25.76, 24.35, 10.17. HRESI-MS *m*/*z* calcd for [M + H]^+^ C_23_H_26_F_2_N_3_O: 398.2038, found: 398.2038.

#### 3.1.12. (5-Chloro-3-methyl-1H-indol-2-yl)(4-phenylpiperazin-1-yl)methanone (**6a**)

Yield % 85, m.p 165–167 °C, ^1^H NMR (400 MHz, CDCl_3_) δ 9.0 (s, 1H, indole NH), 8.25 (d, *J* = 8.8 Hz, 1H, Ar-H), 7.56 (d, *J* = 2.0 Hz, 1H, Ar-H), 7.40 (dd, *J* = 8.7, 2.0 Hz, 1H, Ar-H), 7.35–7.21 (m, 5H, Ar-H), 3.76 (t, *J* = 7.6 Hz, 4H, piperazin-H), 3.00 (t, *J* = 8.0 Hz, 4H, piperazin-H), 2.65 (s, 3H, CH_3_). ^13^C NMR (101 MHz, CDCl_3_) δ 164.41 (C=O), 150.78, 134.46, 129.28, 128.23, 127.44, 125.47, 124.14, 120.80, 119.61, 118.01, 116.80, 112.67, 49.87, 18.26, 14.76. HRESI-MS *m*/*z* calcd for [M + H]^+^ C_20_H_21_ClN_3_O: 354.1368, found: 354.1367.

#### 3.1.13. (6-Chloro-3-methyl-1H-indol-2-yl)(4-phenylpiperazin-1-yl)methanone (**6b**)

Yield % 80, m.p 180–182 °C, ^1^H NMR (400 MHz, CDCl_3_) δ 9.68 (s, 1H, indole NH), 7.47 (d, *J* = 8.5 Hz, 1H, Ar-H), 7.37–7.25 (m, 2H, Ar-H), 7.08 (d, *J* = 8.0 Hz, 1H, Ar-H), 6.95–6.92 (m, 3H, Ar-H), 3.88 (t, *J* = 5.1 Hz, 4H, piperazin-H), 3.22 (t, *J* = 5.3 Hz, 4H, piperazin-H), 2.38 (s, 3H, CH_3_). ^13^C NMR (101 MHz, CDCl_3_) δ 164.69 (C=O), 150.82, 136.51, 129.76, 129.29, 127.52, 126.64, 120.73, 120.67, 120.56, 116.78, 112.10, 111.53, 49.88, 10.18. HRESI-MS *m*/*z* calcd for [M + H]^+^ C_20_H_21_ClN_3_O: 354.1368, found: 354.1368.

#### 3.1.14. (5,7-Difluoro-3-methyl-1H-indol-2-yl)(4-phenylpiperazin-1-yl)methanone (**6c**)

Yield % 82, m.p 171–173 °C, ^1^H NMR (400 MHz, CDCl_3_) δ 9.61 (s, 1H, indole NH), 7.29 (t, *J* = 7.9 Hz, 2H, Ar-H), 7.13–6.66 (m, 5H, Ar-H), 3.89 (t, *J* = 5.8 Hz, 4H, piperazin-H), 3.23 (t, *J* = 5.7 Hz, 4H, piperazin-H), 2.34 (s, 3H, CH_3_). ^13^C NMR (101 MHz, CDCl_3_) δ 163.99 (C=O), 150.78, 129.34, 129.28, 120.79, 116.82, 100.35, 100.31, 100.12, 100.08, 99.28, 99.08, 98.78, 49.94, 10.12. HRESI-MS *m*/*z* calcd for [M + H]^+^ C_20_H_20_F_2_N_3_O: 356.1569, found: 356.1568.

#### 3.1.15. N-Benzyl-5-chloro-3-methyl-1H-indole-2-carboxamide (**7**)

Yield % 84, m.p 203–205 °C, ^1^H NMR (400 MHz, DMSO-*d*_6_) δ 11.38 (s, 1H, indole NH), 8.43 (t, *J* = 5.9 Hz, 1H, amide NH), 7.64 (d, *J* = 2.1 Hz, 1H, Ar-H), 7.41–7.20 (m, 6H, Ar-H), 7.17 (dd, *J* = 8.7, 2.1 Hz, 1H, Ar-H), 4.50 (d, *J* = 5.9 Hz, 2H, NHCH_2_), 2.48 (s, 3H, CH_3_). ^13^C NMR (101 MHz, DMSO-*d*_6_) δ 162.16 (C=O), 139.83, 134.16, 129.66, 129.56, 128.76, 127.82, 127.28, 124.10, 119.41, 113.96, 113.46, 42.92, 10.07. HRESI-MS *m*/*z* calcd for [M + H]^+^ C_17_H_16_ClN_2_O: 299.0946, found: 299.0946.

### 3.2. Biology

#### 3.2.1. In Vitro Anticancer Activity

##### Cell Viability Assay

The MCF-10A (human mammary gland epithelial) cell line was used in the cell viability experiment. Compounds **5a**–**k**, **6a**–**c**, and **7** were incubated with MCF-10A cells for 4 days at 50 µM concentration, and the viability of cells was determined using the 3-(4,5-dimethylthiazol-2-yl)-2,5-diphenyltetrazolium bromide (MTT) test [26]. 

##### Antiproliferative Activity

Using the MTT assay with doxorubicin as the reference drug, the antiproliferative activities of **5a**–**k**, **6a**–**c**, and **7** against four human cancer cell lines, including pancreas cancer cell line (Panc-1), breast cancer cell line (MCF-7), colon cancer cell line (HT-29), and epithelial cancer cell line (A-549) were investigated [27].

#### 3.2.2. EGFR Inhibitory Activity

The inhibitory efficacy of **5d**, **5e**, and **5h**–**k** against EGFR was evaluated using the EGFR-TK assay [28]. 

#### 3.2.3. CDK2 Inhibitory Assay

Compounds **5d**, **5e**, and **5h**–**k** were further examined for their ability to inhibit the CDK2 enzyme [29]. 

#### 3.2.4. Apoptosis Assay

##### Activation of Proteolytic Caspases Cascade

The effects of compounds **5d**, **5e**, and **5h** on caspases 3, 8, and 9 were assessed and compared to doxorubicin, which was used as a control [33]. 

##### Cytochrome C Assay

Indole-2-carboxamide derivatives **5d**, **5e**, and **5h** were evaluated as Cytochrome C activators in the MCF-7 human breast cancer cell line [35]. 

##### Bax and Bcl-2 Levels Assay

The most potent caspase activators, **5d** and **5e**, were investigated for their influence on Bax and Bacl-2 levels in a breast cancer cell line (MCF-7) using doxorubicin as a control [36]. 

##### Effect of Compounds **5d** and **5e** on p53 Transcription in MCF-7

The effects of **5d** and **5e** on p53 transcription were evaluated and compared to doxorubicin as a control [43]. 

## 4. Conclusions

A new series of EGFR/CDK2 dual inhibitors containing indole-2-carboxamides has been reported. A total of fifteen target compounds were synthesized and evaluated in vitro against four cancer cell lines as well as these two kinases. The majority of the compounds examined had promising antiproliferative activity. The most effective of these compounds were **5d**, **5e**, **5h**, **5i**, **5j**, and **5k**. The novel compounds induced apoptosis and increased Caspase 3, 8, 9, and Cytochrome C levels. Furthermore, the investigated compounds increased Bax and p53 levels while decreasing anti-apoptotic Bcl2 protein levels. Following optimization, these compounds form a novel class of compounds capable of acting as potent apoptotic anticancer agents for both EGFR and CDK2.

## Figures and Tables

**Table 1:** Antiproliferative activity of compounds **5a**–**k**, **6a**–**c**, **7**, and **Doxorubicin**.

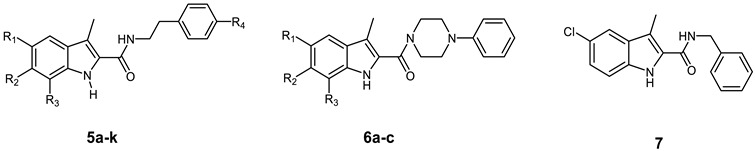						
Compd.	Cell Viability %	Antiproliferative Activity IC_50_ ± SEM (µM)				
		A-549	MCF-7	Panc-1	HT-29	Average
**5a**	89	3.70 ± 0.30	3.20 ± 0.30	3.90 ± 0.30	3.90 ± 0.30	3.70
**5b**	87	3.20 ± 0.30	2.90 ± 0.30	3.50 ± 0.30	3.60 ± 0.30	3.30
**5c**	87	1.70 ± 0.20	1.40 ± 0.20	1.80 ± 0.20	1.80 ± 0.20	1.70
**5d**	89	1.05 ± 0.10	0.90 ± 0.10	1.10 ± 0.10	1.10 ± 0.10	1.05
**5e**	93	0.95 ± 0.05	0.80 ± 0.05	1.00 ± 0.20	1.10 ± 0.10	0.95
**5f**	90	1.90 ± 0.20	1.70 ± 0.20	2.10 ± 0.20	2.10 ± 0.20	1.95
**5g**	89	4.90 ± 0.50	4.80 ± 0.50	5.20 ± 0.50	5.10 ± 0.50	5.00
**5h**	87	1.00 ± 0.10	0.90 ± 0.10	1.20 ± 0.10	1.20 ± 0.10	1.10
**5i**	90	1.55 ± 0.20	1.30 ± 0.10	1.60 ± 0.20	1.65 ± 0.20	1.50
**5j**	83	1.20 ± 0.10	1.00 ± 0.10	1.30 ± 0.10	1.30 ± 0.10	1.20
**5k**	87	1.40 ± 0.20	1.20 ± 0.10	1.50 ± 0.20	1.50 ± 0.20	1.40
**6a**	90	2.90 ± 0.30	2.60 ± 0.20	2.80 ± 0.20	2.90 ± 0.20	2.80
**6b**	91	2.50 ± 0.20	2.30 ± 0.20	2.65 ± 0.20	2.80 ± 0.20	2.60
**6c**	89	2.20 ± 0.20	2.10 ± 0.20	2.40 ± 0.20	2.50 ± 0.20	2.30
**7**	91	4.10 ± 0.40	4.00 ± 0.40	4.40 ± 0.40	4.60 ± 0.40	4.30
Doxorubicin	-	1.20 ± 0.20	0.90 ± 0.10	1.40 ± 0.20	1.00 ± 0.10	1.10

**Table 2:** Effects of compounds **5d**, **5e**, **5h**–**k**, and **Erlotinib** on EGFR and Dinaciclib.

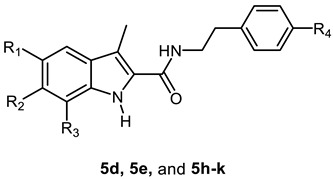						
Compd.	R_1_	R_2_	R_3_	R_4_	EGFR Inhibition IC_50_ ± SEM (nM)	CDK2 Inhibition IC_50_ ± SEM (nM)
**5d**	Cl	H	H	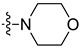	89 ± 6	23 ± 2
**5e**	Cl	H	H	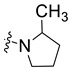	93 ± 8	13 ± 1
**5h**	Cl	H	Cl	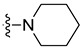	118 ± 10	11 ± 1
**5i**	Cl	H	Cl	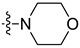	137 ± 12	27 ± 3
**5j**	F	H	F	H	98 ± 8	34 ± 3
**5k**	F	H	F	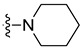	129 ± 11	19 ± 2
Erlotinib	--	--	--	--	80 ± 5	ND
Dinaciclib	--	--	--	--	ND	20 ± 2

**Table 3:** Effects of compounds **5d**, **5e**, **5h** and doxorubicin on active Caspases 3, 8, 9 and Cytochrome C in MCF-7 breast cancer cell line.

Compound Number	Caspase-3		Caspase-8		Caspase-9		Cytochrome C	
	Conc (pg/mL)	Fold Change	Conc (ng/mL)	Fold Change	Conc (ng/mL)	Fold Change	Conc (ng/mL)	Fold Change
**5d**	570.00 ± 5.00	8.70	1.94	9.70	16.90	17.80	0.70	14
**5e**	635.50 ± 5.50	9.70	2.17	10.90	17.25	18.15	0.80	16
**5h**	537.50 ± 5.00	8.20	1.88	9.50	16.65	17.50	0.65	13
Doxorubicin	503.50 ± 4.50	7.70	1.80	9.00	16.25	17.00	0.60	12
Control	65.50	1	0.20	1	0.95	1	0.05	1

**Table 4:** Effects of compounds **5d**, **5e**, and doxorubicin on Bax and Bcl-2.

Compd. No.	Bax		Bcl-2	
	Conc (pg/mL)	Fold Change	Conc (ng/mL)	Fold Change
**5d**	289.70 ± 2.50	35	0.89	5.70
**5e**	296.50 ± 2.50	36	0.87	5.90
Doxorubicin	275.80 ± 2.50	33	0.98	5.20
Cont.	8.25	1	5.10	1.00

**Table 5:** Effects of compounds **5d**, **5e**, and doxorubicin on p53.

Compd. No.	p53	
	Conc (pg/mL)	Fold Change
**5d**	1375 ± 15	27
**5e**	1435 ± 15	28
Doxorubicin	1265 ± 10	25
Cont.	51.50	1

**Table 6:** MD of compounds **5d** and **5e** within EGFR and CDK2 active sites.

Compd.	EFGR (PDB ID: 1M17)					CDK2 (PDB ID: 1PYE)				
	S ^a^	RMSD (Å)	Binding Interactions			S	RMSD (Å)	Binding Interactions		
			a.a. Residue	Type	Distance (Å)			a.a. Residue	Type	Distance (Å)
**5d**	−6.90	1.49	THR 766	H-acceptor	2.91	−6.03	1.77	GLN 131	H-donor	3.60
			LEU 694	pi-H	3.99					
**5e**	−6.79	1.51	LEU 694	pi-H	3.70	−6.99	1.68	LYS 33	pi-cation	4.65
Ref	−7.3 ^b^	1.28	GLN 767	H-donor	3.15	−5.89 ^c^	1.84	GLU 81	H-donor	3.05
			MET 769	H-acceptor	2.70			LEU 83	H-acceptor	3.07

^a^ S: docking score (kcal/mol); ^b^ Ref: co-crystallized ligand (Erlotinib); ^c^ Ref: co-crystallized ligand (Dinaciclib).

## Data Availability

Data is contained within the article.
